# Study on Improved Flight Coefficient Estimation and Trajectory Analysis of a Flying Disc through Onboard Magnetometer Measurements

**DOI:** 10.3390/s18103564

**Published:** 2018-10-20

**Authors:** Juhwan Lee, Byungjin Lee, Jin Woo Song, Young Jae Lee, Sangkyung Sung

**Affiliations:** 1 Department of Aerospace Information Engineering, Konkuk University, Seoul 05029, Korea; lmkknjjb@naver.com (J.L.); schumir@konkuk.ac.kr (B.L.); younglee@konkuk.ac.kr (Y.J.L.); 2 School of Intelligent Mechatronic Engineering, Sejong University, Seoul 05006, Korea; jwsong@ieee.org

**Keywords:** flying disc, magnetic sensor, rotational rate, yaw damping, flight trajectory, embedded sensor

## Abstract

This paper proposes a novel and accurate method for estimating the flight coefficient of a flying disc typically operating at a high rotation rate. In particular, the proposed method introduces a new algorithm that takes advantage of magnetic data measured by a miniaturized sensor module onboard a conventional disc. Since the geomagnetic field measured by the magnetic sensor mounted on the rotating body yields a general sinusoidal waveform, a frequency domain analysis is employed in computing the rotational rate. Furthermore, on the basis of the estimated rate during a whole flight period, a yaw damping derivative coefficient is derived, which enables an accurate prediction of the disc’s flight trajectory. For performance verification, both a reference rotation table test and a real flight test are performed, for which a miniaturized embedded sensor module is designed and manufactured for an onboard flight test. A reference rotation test validates the performance of the proposed method. Subsequently, a flight test, in which a simulator-based trajectory is compared with the true reference trajectory, verifies that the proposed method better predicts the flight trajectory by incorporating the estimated coefficient.

## 1. Introduction

This paper presents a new method for estimating the flight coefficient of a fast rotating flying disc using geomagnetic field information acquired from an onboard sensor module. To achieve accurate flight characteristics of the disc such as its flying trajectory, velocity, and attitude, various inertial data and other auxiliary sensor measurements are required. Especially, this study focuses on obtaining the yaw rate and yawing moment coefficient, which play a significant role in the flight governing dynamics of a disc. Despite its importance in determining the flight states of a disc, the yawing moment coefficient is relatively less investigated than other flight coefficients. The reason for this mainly originates from the difficulty in measuring the fast rotating rate in the yaw axis, since a disc yaw rate mostly exceeds the dynamic range of conventional onboard sensors.

In previous researches, various techniques have been applied to estimate the aerodynamic coefficient and to analyze the flight characteristics of a flying disc. Recently, the computational fluid dynamics (CFD) analysis technique has been proposed but it presents a burdensome computational complexity. In reference [1], the calculation of the lift, drag, and pitching moment coefficients is carried out by assuming a static state, in which the influence of rotation can be neglected. Then, the computation of the lateral force, rolling moment, and yawing moment, which are sensitive to the rotational rate change, is attempted by considering an air flow model equation in a constrained flight condition [2]. In addition, some pioneering studies applying the computational fluid analysis method to characterize a flying disc in detail have been reported, which focus on estimating the flight coefficient considering the ground effect and disc shape [3,4]. However, most studies based on CFD provided only preliminary results because of a limited grid density, imperfect modeling in the computational method, and lack of further experimental verification. Unlike the computational fluid analysis, the wind tunnel method attempted to estimate the flight coefficient through an experimental method. A mechanism to actually rotate the flying disc was implemented inside the wind tunnel, such that it could change the flow velocity and angle of attack, to estimate the flight coefficient [5]. However, only horizontal flight coefficients, including rolling and pitching moment, were reported with respect to the angles of attack and advance ratio because of the inevitable experimental constraints within the wind tunnel.

Even though these studies provide a concrete background for characterizing a disc’s flight dynamics, further investigation is required depending on the flight coefficient type. Specifically, static coefficients such as lift, drag, and moment coefficients in horizontal axes have been treated in depth. Also, dynamic flight coefficients in the roll and pitch axis have been provided under specific rotating conditions. However, for a more sophisticated analysis of the flight characteristics and trajectory prediction, it is essential to study the rotational moment characteristics for the vertical axis, i.e., the yaw moment coefficient. This is because the dynamic coefficient related to the yawing moment plays a significant role in determining the flight trajectory, especially during the latter periods of the flight. Note that the terminal flight trajectory highly relies on the roll stability, while the principal roll moment coefficient is coupled with the yaw rate. Thus, the yaw rate transient characteristics serve as a governing factor for roll attitude determination. Consequently, the terminal trajectory shape is greatly affected by the yaw damping derivative curve, which motivates to devise an accurate method for yaw rate computation.

Generally, the yaw rate during flight periods is very high, thus only limited methods can be applied to measure accurately the yawing moment coefficient of a rotating disc. For computing the yaw rate, a previous study employed visual measurements with the help of a high-speed camera, which in turn has limited measurement ranges due to its spatial constraints [6]. The accelerometer-based coefficient estimation technique is suggested as an alternative method, which approximates the rotational rate via centripetal acceleration measurements [7,8]. Even though these methods provided fair estimation results without spatial constraints on the flight experiment, they simultaneously showed performance degradation when the sensor installment is not ideally implemented. Because of the disc’s high rotational rate properties, the computation result is very sensitive to displacements of the sensor location from the ideal center of disc. Thus, even a very small axial misalignment between the accelerometer and the center of rotation generates a large deviation in the yaw rate estimation, which may require a burdensome compensation process. Besides, the measured acceleration is very susceptible to undesired disturbances like gravity, acceleration of the rotating origin, angular accelerations, and other inertial noises.

Meanwhile, a representative measurement type independent from the inertial force is a magnetic field, which is commonly applied to attitude determination systems, indoor positioning, localization, and tracking algorithms, and integrated navigation systems. For instance, a geomagnetic measurement is adapted in satellite’s robust attitude determination problem under a measurement malfunction scenario [9]. A preliminary result for indoor positioning is presented by designing a cluster of pulsed magnetic signals to obtain relative distances [10]. Alternating magnetic measurements generated from multiple source coils are used for computing the distance from the transmitter node to the receiver node, and then a trilateration method is applied for localization [11]. Recently, the study was extended to cover orientation as well as 3D localization under a spatial test region [12]. Spectral analysis of magnetic measurement was also performed for estimating the compensation coefficients of a fluxgate sensor via the harmonic decomposition method [13], for identifying the orbit semi-major axis regardless of satellite attitude [14], and for designing an invasive diagnosis method to detect broken rotor bars in large induction machines [15]. Although relevant works are scarce, the frequency domain analysis of magnetic data is expected to have wider applications because of its advantage of allowing measurement unaffected by inertial disturbance, wide bandwidth, and the penetrating characteristics of a magnetic field.

In this background, the present study suggests a new method to obtain the yaw rate of a fast rotating object and subsequently to estimate the yawing moment coefficient based on non-inertial sensor measurements. First, an algorithm for providing the yaw rate is developed, which employs a moving horizon fast Fourier transform (FFT) analysis. For the experimental verification, a miniaturized onboard sensor module is designed and manufactured to store the flight test data. Specifically, a low-cost microelectronic mechanical system (MEMS) magnetometer is used for measuring alternating geo-magnetic fields during the disc flight. For performance comparison, the estimated yaw rate is analyzed with a comparative rate measurement system and a reference rate table. Furthermore, to demonstrate the validity of the proposed method, the estimated coefficient is applied to a disc flight simulator implementing the disc aerodynamics model. In the simulator, other primary coefficients, such as lift, drag, and pitching/rolling moment, are adopted from the reference parameters of previous studies and the CFD analysis results [2,3,4]. In this way, the simulator-based trajectory obtained from repeated flight test data demonstrates the validity of the presented coefficient estimation algorithm. In fact, the true reference flight trajectory is acquired from a GPS receiver onboard the sensor module, and the comparative accelerometer-based prediction is plotted for performance comparison. The rest of the paper is organized as follows. Section 2 presents the estimation methods of the high angular rate; Section 3 illustrates performance verification through a reference rate test; Section 4 presents the application of the algorithm to the disc dynamics through a flight test and a simulation study; conclusions are presented in Section 5.

## 2. Estimation Method of the High Angular Rate

In this section, a new algorithm to estimate the inflight high angular rate of a disc is presented. The proposed rate estimation is based on the non-inertial measurement by a magnetometer, which can avoid undesired errors caused by methods performing inertial measurements. For a comparative study, a previous work computing the rotation rate using an onboard accelerometer [7] was revisited. Additionally, in the acceleration-based approach, an error analysis is newly presented to quantify its estimation performance. Finally, a rotation test is done using the 1-axis precision rate table and the prototype bulk sensor module, which demonstrates the improved performance of the proposed method.

### 2.1. Rate Estimation Using the Accelerometer

**Figure 1:**
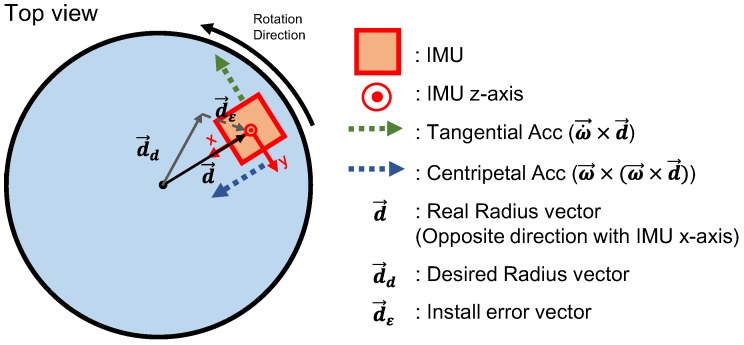
Configuration and geometry of the sensor module mounted on a disc. IMU: inertial measurement unit.

First, the acceleration-based method for measuring the high yaw rate onboard the disc, as reported in reference [7] is revisited. The basic principle of the presented method is to use a centripetal acceleration to compute the rotation rate. In this study, an error equation is further developed to evaluate the estimation performance of the accelerometer-based scheme. Figure 1 shows the geometry of the sensor module mounted on a rotating disc and the parameters denoting installation errors.

Equation (1) describes the total acceleration term measured by the accelerometer on the disc:

(1) $$\vec{a}=\ddot{\vec{R}}+\vec{\omega }\times (\vec{\omega }\times \vec{d})+\dot{\vec{\omega }}\times \vec{d}+\vec{g}$$

where $\ddot{\vec{R}}$ is the acceleration of the B-frame origin, $\vec{\omega }\times (\vec{\omega }\times \vec{d})$ is the centripetal acceleration, $\dot{\vec{\omega }}\times \vec{d}$ is the tangential acceleration, and $\vec{g}$ is the gravity. The centripetal acceleration and the tangential acceleration are the dominant terms caused by the fast rotational motion, while the translation and gravity terms are less influential. In Equation (1), note that the distance from the center of the disc rotation to the measurement center of the accelerometer $\vec{d}$ affects directly the measured acceleration. In Figure 1, the actual inertial measurement unit (IMU) position vector $\vec{d}≜{\left[d_{x}\;d_{y}\;d_{z}\right]}^{T}$ is modeled according to Equation (2):

(2) $$\vec{d}={\vec{d}}_{d}+{\vec{d}}_{\varepsilon }$$

where ${\vec{d}}_{d}≜{\left[d_{dx}\;d_{dy}\;d_{dz}\right]}^{T}$ denotes an ideal IMU position vector from the disc center, and ${\vec{d}}_{\varepsilon }≜{\left[d_{\varepsilon x}\;d_{\varepsilon y}\;d_{\varepsilon z}\right]}^{T}$ denotes the installation error vector.

Next, we consider the practical accelerometer output model that contains nonlinearity, cross-axis sensitivity, bias, and random noise [16]. The measured acceleration can be modeled according to Equation (3):

(3) $${\vec{a}}_{{}_{M}}=(I+S_{A})\vec{a}+C_{A}\vec{a}+{\vec{b}}_{A}+{\vec{w}}_{A}$$

where *S_A_* is a diagonal nonlinearity matrix, *C_A_* is a cross-axis sensitivity matrix, ${\vec{b}}_{A}$ is the bias vector, ${\vec{w}}_{A}$ is the random noise vector. Then, a new sensitivity matrix can be defined according to Equation (4):

(4) $$S_{T}≜(I+S_{A})+C_{A}=\left[\begin{array}{ccc} 1+S_{Ax} & S_{xy} & S_{xz}\\ S_{yx} & 1+S_{Ay} & S_{yz}\\ S_{zx} & S_{zy} & 1+S_{Az} \end{array}\right]$$

where $S_{ij}$ denotes the coupling factor between the *i* and *j* axes. Thus, Equation (3) is simplified as:

(5) $${\vec{a}}_{{}_{M}}=S_{T}\vec{a}+{\vec{b}}_{A}+{\vec{w}}_{A}$$

where ${\vec{b}}_{A}$ is the accelerometer’s bias, ${\vec{w}}_{A}$ is the accelerometer’s white noise. Using Equations (1) and (5), the *x*-axis component can be obtained in Equation (6):

(6) $$\begin{array}{ll} a_{M,x}= & \left(1+S_{Ax})[{\ddot{R}}_{x}-(q^{2}+r^{2})d_{x}+(pq-\dot{r})d_{y}+(pr+\dot{q})d_{z}+g_{x}\right]\\ & +S_{xy}[{\ddot{R}}_{y}-(p^{2}+r^{2})d_{y}+(pq+\dot{r})d_{x}+(qr-\dot{p})d_{z}+g_{y}]\\ & +S_{xz}[{\ddot{R}}_{z}-(p^{2}+q^{2})d_{z}+(pr-\dot{q})d_{x}+(qr+\dot{p})d_{y}+g_{z})]+b_{Ax}+w_{Ax} \end{array}$$

where the angular rate in the body frame is given by $\vec{\omega }={\left[p\;q\;r\right]}^{T}$. Since the disc rotates very fast in the *z*-axis, Equation (6) can be reduced to:

(7) $$a_{M,x}\approx (1+S_{Ax})({\ddot{R}}_{x}-r^{2}d_{x}+g_{x})+S_{xy}({\ddot{R}}_{y}-r^{2}d_{y}+g_{y})+S_{xz}({\ddot{R}}_{z}+g_{z})+b_{Ax}+w_{Ax}$$

where $r^{2}\gg p^{2}≅q^{2}≅pq≅\dot{r}≅0$ is assumed. Note that a negative sign is assigned to *d_x_* and *d_y_* in Equation (7), since the distance vector is defined as the opposite direction of IMU’s *x*-axis. Rearranging Equation (7) in terms of yaw rate generates the following equation:

(8) $$r=\sqrt{\frac{a_{M,x}-[(1+S_{Ax})({\ddot{R}}_{x}+g_{x})+S_{xy}({\ddot{R}}_{y}+g_{y})+S_{xz}({\ddot{R}}_{z}+g_{z})+b_{Ax}+w_{Ax}]}{-d_{dx}-[S_{Ax}d_{dx}+(1+S_{Ax})d_{\varepsilon x}+S_{xy}(d_{dy}+d_{\varepsilon y})]}}$$

In Equation (8), the numerator contains the cross-axis coupling errors due to linear acceleration and gravity, as well as bias and noise, while the denominator contains radial error components due to the installation error. As the scaled installation error term ($(1+S_{Ax})\;d_{\varepsilon x}+S_{xy}\cdot d_{\varepsilon y}$) perturbs the ideal distance in the denominator ($d_{dx}$), the computed rate can significantly deviate from zero installation error. Intuitively, this effect gets larger when the $d_{dx}$ gets smaller. On the other hand, the error effect from the numerator depends on the rotation condition. When the measured acceleration ($a_{M,x}$) is dominantly derived from the centripetal acceleration (sufficiently large over 2–3 g) with a fast rotation rate, the perturbation effect gets relatively smaller. However, note that a non-zero rate estimate value can appear because of the cross-coupled bias and gravity terms, even with zero rotation rate. Developing Equation (8) further yields:

(9) $$r=\sqrt{\frac{a_{M,x}-\varepsilon_{n}}{-d_{dx}-\varepsilon_{d}}}$$

where the overall error terms are arranged as $\varepsilon_{n}≜\left(1+S_{Ax}\right)\left({\ddot{R}}_{x}+g_{x}\right)+S_{xy}\left({\ddot{R}}_{y}+g_{y}\right)+S_{xz}\left({\ddot{R}}_{z}+g_{z}\right)+b_{Ax}+w_{Ax}$ and $\varepsilon_{d}≜S_{Ax}d_{dx}+\left(1+S_{Ax}\right)d_{\varepsilon x}+S_{xy}\left(d_{dy}+d_{\varepsilon y}\right)$. Assuming the accelerometer is mounted in a plane such that the *z*-axis is exactly aligned with the gravity vector, the uncertainty $\varepsilon_{n}$ can be further simplified as in Equation (10):

(10) $$\varepsilon_{n}=(1+S_{Ax})({\ddot{R}}_{x})+S_{xy}({\ddot{R}}_{y})+S_{xz}({\ddot{R}}_{z}+g)+b_{Ax}+w_{Ax}$$

Using Equation (9), a quantitative analysis predicting the rate estimation uncertainty is developed. Considering the rotational axis and the definition of ${\vec{d}}_{d}$ and ${\vec{d}}_{\varepsilon }$, the uncertainty bound of Equation (9) can be computed as:

(11) $$\sqrt{\left(\frac{a_{M,x}-\varepsilon_{n,\max}}{-d_{dx}-\varepsilon_{d,\min}}\right)}\leq r\leq \sqrt{\left(\frac{a_{M,x}-\varepsilon_{n,\min}}{-d_{dx}-\varepsilon_{d,\max}}\right)}$$

Finally, assuming the error terms in Equation (9) are all neglected, the yaw rate can be simplified into:

(12) $$r=\sqrt{\frac{a_{M,x}}{-d_{dx}}}$$

which is the same as the rate approximation presented in reference [7]. Again, note that the denominator in Equation (12) is positive, as *d_dx_* is always negative for the sensor axis definition in the coordinate system as shown in Figure 1.

### 2.2. Rate Estimation via Magnetic Measurement

Despite the simplicity of the equation for rate estimation in the previous study, it is observed that various sources, including both deterministic and random errors, degrade the estimation accuracy, introducing uncertainties. To overcome the performance degradation under an inertial measurement scheme, this paper proposes a high rate estimation method using magnetometer measurements. In particular, the investigated method attempts to remove the estimation error that is very sensitive to the sensor installation and other error factors propagated from the sensor noise characteristics.

**Figure 2:**
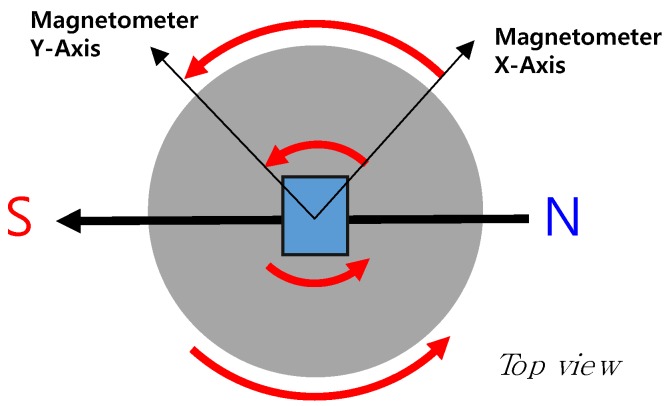
Scheme of the concept for estimating the flying disc’s rotational speed using a magnetometer.

Figure 2 shows a conceptual diagram of the proposed method. Since the geomagnetism is virtually constant around a local ground, the measurement value of the magnetometer onboard the disc takes a sinusoidal waveform during the disc’s flight. That is, the sinusoidal period of the geomagnetism is equal to the rotation period of the flying disc. This principle has the advantage of measuring the rotation rate accurately, regardless of the mounting position or attitude of the embedded sensor module. Also, it is notable that the measurement is entirely separated from the undesired inertial interferences during the flying periods.

**Figure 3:**
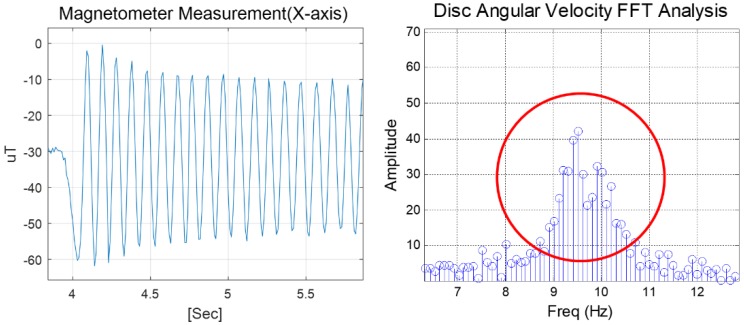
Magnetometer measurement onboard the flying disc (**left**) and overall frequency distribution during flight periods (**right**).

Figure 3 shows an example of geomagnetic data measured by the magnetometer (left) and the resulting frequency characteristics through FFT analysis (right) during whole flying periods. The dominant frequency ranges between 9 and 10 Hz, which implies that the flying disc is rotating at an angular rate of 3240–3600 °/s. As observed from the figure, a frequency uncertainty exists, owing to the spin-down effect during the disc’s flight period. For computing the flight coefficient of the disc, however, it is required to analyze the time-varying characteristics of the angular rate.

**Figure 4:**
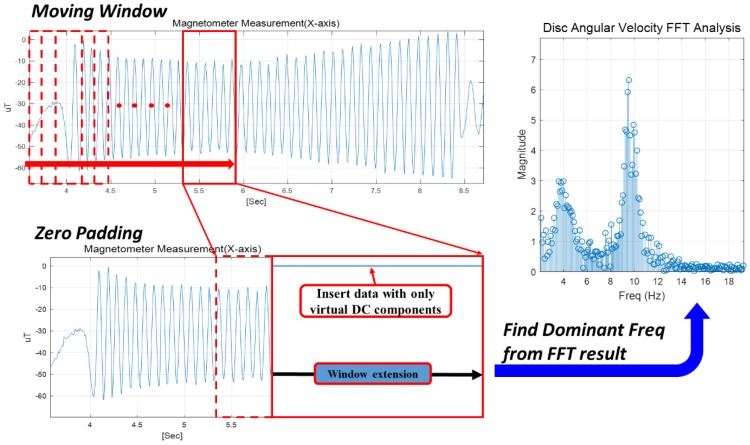
Conceptual block diagram of the magnetometer-based rate estimation algorithm.

To analyze the angular rate on the time-axis, a moving window-based FFT analysis method was applied [17]. Depending on the sampling frequency of the measured data, the window size of the magnetic data was determined for computing the dominant rotation rate at each time instance. Then, by shifting the window gradually, the rotational rate of the disc was sequentially estimated with respect to each period [18]. In practice, when estimating the angular rate using the moving window FFT, zero padding was implemented to increase the frequency resolution sufficiently [19]. Figure 4 shows the block diagram illustrating the presented algorithm.

In summary, the following computing procedure illustrates the proposed rate estimation algorithm based on the moving-window FFT method with magnetometer measurments.

(P-1) Set the sampling Hz (DAQ Hz) and window size *n*. The recommended window size is *n* = 128 when the magnetometer data are acquired at 100Hz.
(P-2) Set the zero component data size *k* (recommended 2^14^ − *n*) for zero padding.
(P-3) Specify the starting point and attach the zero component data to the magnetometer data with length *n*. These attached *n + k* data in a window are called ‘padded data’.
(P-4) Perform the FFT on the padded data and get the amplitude. Determine the frequency vector as below (PE1). The computed amplitude and frequency have the same vector size, and all components are matched. For example, amplitude (1) matches frequency (1).
  (PE1) Freq=[0, 1, 2, …, n+k]×Sampling Hz/(n+k)
(P-5) Find the maximum value of absolute amplitude and get the order of maximum value. If ‖amplitude(j)‖ is maximum, the order is *j*.
(P-6) Finally, calculate the angular velocity by matching the order to the frequency as below (PE2):
  (PE2) Angular veolcity r=Freq(j)×360[°/s]
(P-7) Repeat steps (P-3) to (P-6) while shifting the window gradually.

## 3. Performance Verification through the Reference Rate Test

**Figure 5:**
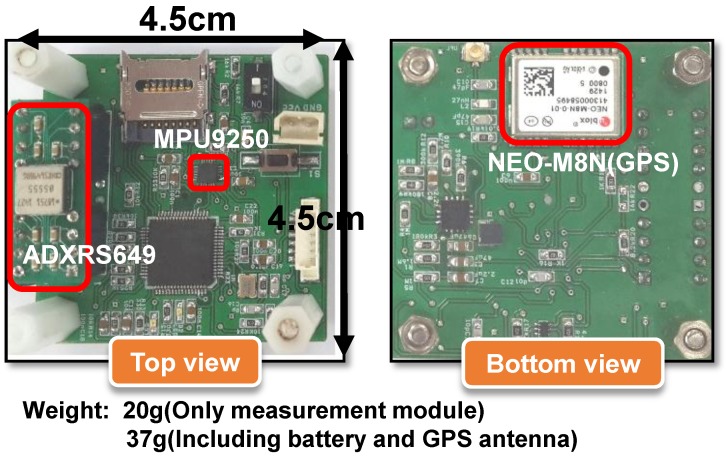
Prototype embedded module with reference sensor.

To verify the rate estimation performance of both methods, a prototype sensor module was fabricated as shown in Figure 5. The top and bottom layout of the manufactured prototype sensor module on a PCB board can be observed in the figure. In particular, a typical MEMS IMU sensor, MPU 9250, made by Invensense Co. Ltd. (San Jose, CA, USA), was chosen to measure the inertial components of acceleration and angular rate as well as the magnetic data. An essential shortcoming for using a conventional IMU sensor is that the dynamic range is hard-limited (in case of MPU 9250, gyro saturation is observed at 2000 °/s.) In practice, most gyroscopes in a 6-DOF or 9-DOF off-the-shelf MEMS IMU have a dynamic range lower than 2000 °/s. MPU 9250 also provides three-axis magnetic data, and the resolution of the embedded magnetometer is 6 m Gauss. It is notable that, in Figure 5, a very exceptional single-axis rate sensor (ADXRS 649, Analog Device Inc., Norwood, MA, USA) with an ultra-high dynamic range is employed in the manufactured prototype sensor module. ADXRS 649 is a bulk-type single axis gyroscope with a dynamic range up to 20000 °/s, used as a comparative reference sensor. Despite its exceptional dynamic range capacity, this bulk-type single-axis gyroscope provides less accurate rate information and, moreover, cannot be properly integrated in a miniaturized onboard module because of its bulky mass and volume. Besides, the GPS receiver, NEO-M8N (Ublox, Thalwil, Switzerland), was included for generating a true reference trajectory. For storing the sensor data, an embedded microprocessor with SD memory was installed.

**Figure 6:**
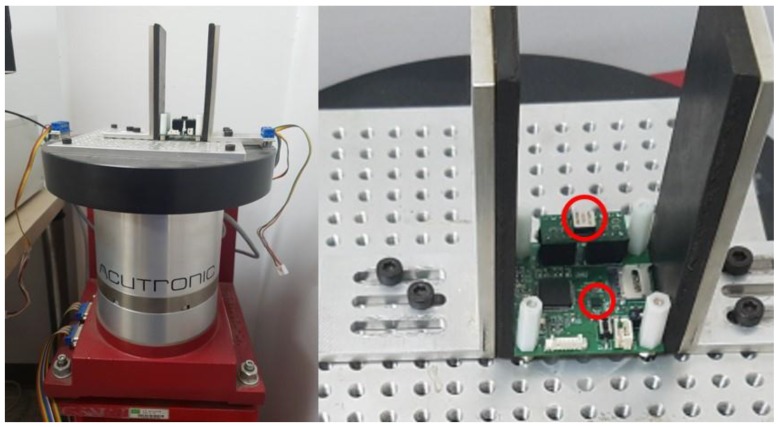
Sensor module mounted on the reference rate table for the performance test.

**Figure 7:**
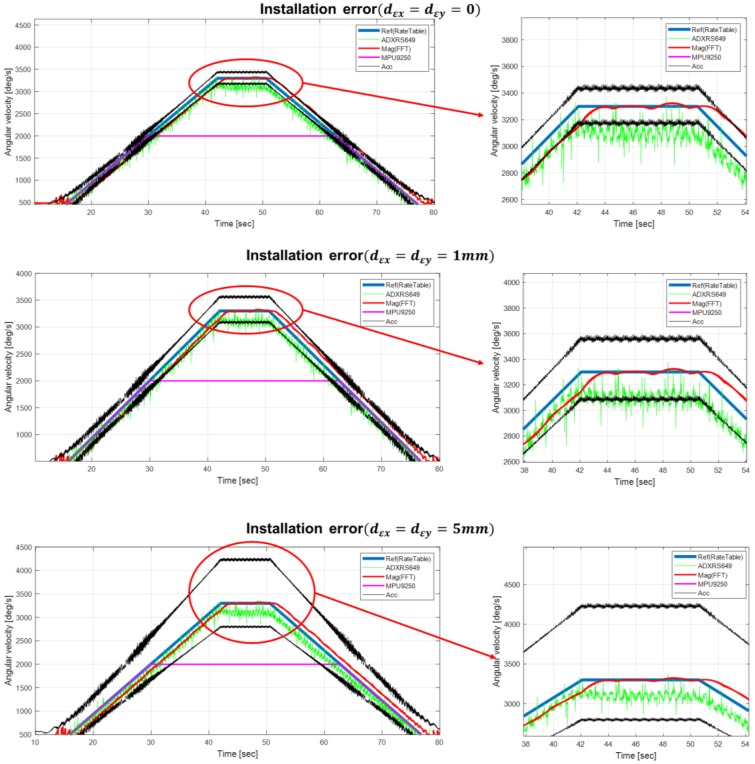
Performance comparison of the rate estimation methods via the rate table test.

To compare the suggested algorithms, an accurate rate table experiment was performed. Figure 6 shows the picture of the experimental setup on a rate table with the prototype sensor module installed. The reference rate table was the Acutronic’s AC1120S, whose rate accuracy is up to 0.001% of the applied rate. Figure 7 demonstrates the rate estimation performance of the proposed method compared with other methods. In Figure 7, the magenta solid line is the measurement result of the angular rate of the MPU-9250, and it can be observed that saturation occurs in the high rate region. On the other hand, the green solid line is the measurement from ADXRS649, which does not saturate because of its wide dynamic range. However, the analog output of ADXRS649 contains notable white noises. Furthermore, since the parameter provided in the data sheet, i.e., the sensitivity for the output correction, is probabilistic, a calibration process using a rate table is essentially needed for an accurate application [20]. On the other hand, the solid black lines show the upper and lower envelopes of the rate estimation result, which reflect various error factors influencing the accelerometer measurement. For a quantitative analysis of the experiment, the accelerometer’s inherent error sources (i.e., nonlinearity, cros-axis sensitivity, bias, random white noise) were assumed at their greater case values. The installation error appeared to dominate; thus, two practical cases were considered, with errors of 1 mm ($d_{\varepsilon x}=d_{\varepsilon y}=0.001)$ and 5 mm ($d_{\varepsilon x}=d_{\varepsilon y}=0.005)$ for each axis.

First, the accelerometer’s inherent error sources were considered, and conservative bounds of each error source were applied to Equation (8). Note that there was no acceleration of the B-frame origin (i.e., $\ddot{R}=0$ in Equation (1)), since it was obtained from a rate table experiment. Each error characteristics of the accelerometer is referred to the specification of MPU-9250; thus, the nonlinearity and cross-axis sensitivity in each axis allowed maximum uncertainties of 0.5% and 2%, respectively. Thus, in this experiment, the worst case of Equation (4) can be presented as:

(13) $$S_{T}^{+}=\left[\begin{array}{ccc} 1.005 & 0.02 & 0.02\\ 0.02 & 1.005 & 0.02\\ 0.02 & 0.02 & 1.005 \end{array}\right]\;$$

Also, the accelerometer bias had a maximum uncertainty of 6% in the horizontal axis and 8% in the vertical axis. Random white noise in each axis had 0.3% uncertainty with respect to gravity. For a conservative analysis, the worst-case uncertainty is applied to the numerator and denominator terms of Equation (8), where the sensor’s specified error distribution resulted in an estimation envelope. Even though the inherent sensor characteristics decreased the rate determination accuracy, the estimation performance was more dominantly governed by the installation error present in the denominator term $\varepsilon_{d}$. For illustrating the estimation performance corresponding to different installing errors, results from two cases are presented. The *x*-axis installation error ($d_{\varepsilon x})$ was applied deliberately with the ratio of 6.2% (1 mm) and 31% (5 mm), respectively, from the actual IMU position’s *x*-axis ($d_{dx}=16.1\;\mathrm{mm})$. In Figure 7, the second subplot shows the estimation result when assuming a 1 mm installation error, and the third subplot is the result when assuming a 5 mm installation error. As observed in Equation (8), the installation error significantly decreased the rate estimation accuracy, which highly depends on the relative distance ratio. As the installation error approached zero, the upper and lower uncertainty bound decreased around the true rate region. Note that, even with zero installation error, the uncertainty band does not converge to zero since the worst-case perturbation from the cross-axis coupled terms are still present in the denominator of Equation (8). Consequently, when the sensor module is actually mounted on the disc, the sensor module should be placed as far from the center of rotation as possible in order to minimize the cross-axis perturbation effect, while, simultaneously, the induced centripetal acceleration should be within the input range of the accelerometer.

Finally, the red solid line in Figure 7 represents the estimated angular rate of the proposed algorithm when using a magnetometer. Compared with other methods, the rate estimation of the proposed algorithm provides the most accurate result, free from mechanical interferences, misalignment, and electrical noises. It is also revealed through repeated tests that the presented method provides consistency in the estimation performance, even with a distributed location of the sensor module. Furthermore, note that the proposed method has the advantage that a calibration procedure of the magnetometer’s raw data is not required, since an offset or scale factor mismatch does not affect the spectral distribution of the magnetic measurement data. This implies that a tedious sensor calibration process to remove the hard- and soft-iron effect is unnecessary. In conclusion, despite a small distortion in the rate estimate result, the mean accuracy during the overall estimation periods proved to be superior to that of other comparative methods in the high angular rate region.

[Remark] Despite its accuracy of the rate estimation in the high angular rate region, some drawbacks were observed. First, a small distortion on the estimation result, even at a static rate input, was observed, as shown in the enlarged portion of Figure 7. In fact, since a low-grade magnetometer was embedded in the MPU-9250 chipset, its bandwidth was slow, and the sampling rate was not sufficient for providing alternating geomagnetic measurements precisely. Also, the embedded microprocessor had limited computing performance, thus a noticeable magnetic data loss was observed. Consequently, these measurement imperfections of the onboard magnetometer induced a distortion in the estimation results. From a computational viewpoint, a spectral analysis may increase the computational burden compared with the acceleration-based method. Also, a slight delay in the estimation results is observed in Figure 7, which was caused by the moving-window approach in implementing the FFT algorithm. For offline applications, this delay can be compensated through shifting the estimation result by half of the window period. Besides, to achieve spectral separation effectively from low-frequency band interferences, a threshold yielding an effective estimation range was employed. With this threshold, the proposed method generically possesses a limited estimation performance in the low rotation rate region. In the implementation for disc analysis, a threshold of 500 °/s was designed considering the typical flight conditions of the disc. Lastly, a real-time implementation was not applied in the present work, thus an improved embedded sensor platform and computing optimization are left for further investigation.

## 4. Flight Test and Application to a Disc Flight Dynamics Simulation

In this section, the proposed rate estimation method is applied to realize a disc flight dynamics simulation and verified through a real flight test.

### 4.1. Manufacture of a Miniaturized Module and Performance Evaluation

**Figure 8:**
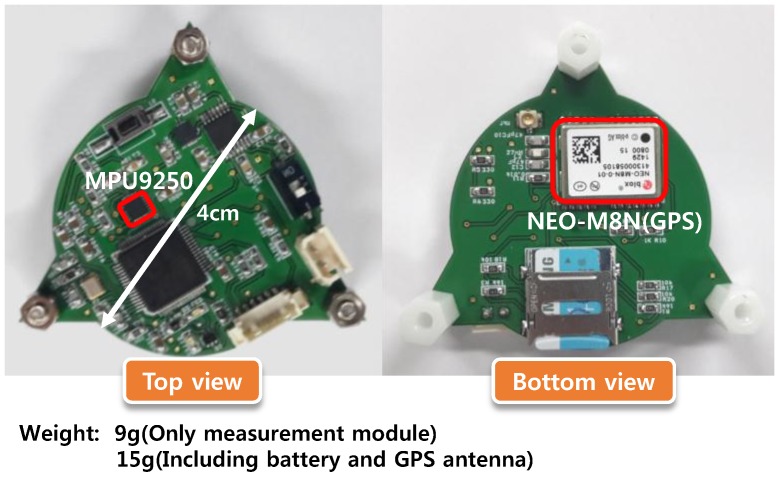
Miniaturized onboard sensor module (including 3-DoF IMU, magnetometer, GPS receiver, SD memory, and battery).

**Figure 9:**
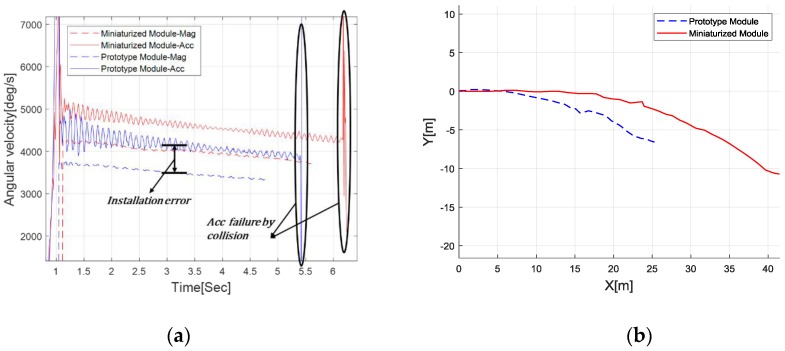
Module’s performance evaluation measuring the flying disc’s (**a**) angular rate and (**b**) flight trajectory.

To achieve the minimum loading effect on the disc flight characteristics, a miniaturized embedded sensor module was designed and tested. Thus, a bulky ADXRS649 was removed, and the circuit layout was improved for lighter onboard implementation. Figure 8 shows the photograph of a manufactured sensor module. The shape changed from square to circle, and the weight of the module itself was reduced by 9 g. In addition, the weight of the subcomponents, such as battery and GPS antenna, were further reduced. Thus, the total weight (15 g) was reduced to less than half of that of the previous sensor module (37 g) using the miniaturized module. Note that the weight of the new sensor module was less than 10% of the net disc weight (164 g). The miniaturized module’s performance evaluation was suggested by the angular rate and trajectory, as shown in Figure 9. The rate was computed through the proposed algorithm, while the trajectory was obtained through the GPS receiver. As noted in the previous section, the rate estimation had an observable sinusoidal perturbation due to the imperfect sensor measurement, yet the mean accuracy iwa proven through the reference rate table test. In repeated throwing conditions, the new sensor module (red dashed line) yielded a higher rotational rate (about 4200 °/s initially), which was 10% higher than that of the previous sensor module (blue dashed line). Also, it was observed that the flight time and distance of the miniaturized module onboard disc were 30% larger as a result of the reduction of the total weight and drag effect.

### 4.2. Disc Flight Dynamics

In this section, the disc dynamic model is briefly introduced to relate the aerodynamic coefficient to the estimated rotational rate. First, consider the aerodynamic force and moment equations presented using the aerodynamic coefficients, as shown in Equation (14). Note that ρ is the air density, *V_T_* is the magnitude of the velocity vector, *A* is the flying disc’s planform area, *d* is the disc’s diameter, *L* and *C_L_* are the lift and lift coefficients, *D* and *C_D_* are the drag and drag coefficients, *Y* and *C_Y_* are the side force and side force coefficients, *R* and *C_R_* are the rolling moment and rolling moment coefficients, *M* and *C_M_* are the pitching moment and pitching moment coefficients, and *N* and *C_N_* are the yawing moment and yawing moment coefficients. Note that the side force *Y* is assumed to be zero because of the symmetrical shape of the disc [21].

(14) $$\begin{array}{cc} \begin{array}{l} L=\frac{1}{2}\rho V_{T}^{2}AC_{L},\\ D=\frac{1}{2}\rho V_{T}^{2}AC_{D},\\ Y=\frac{1}{2}\rho V_{T}^{2}AC_{Y}≃0, \end{array} & \begin{array}{l} R=\frac{1}{2}\rho V_{T}^{2}AdC_{R}\\ M=\frac{1}{2}\rho V_{T}^{2}AdC_{M}\\ N=\frac{1}{2}\rho V_{T}^{2}AdC_{N} \end{array} \end{array}$$

In previous reports, it was shown that the lift, drag, and pitching moment coefficients of the disc are not virtually affected by the rotational rate and, thus, can be approximated as static coefficients [5]. Generally, *C_L_*, *C_D_*, and *C_M_* are computed as a function of *α*, yet a detailed description is skipped as it is out of the scope of this paper. On the other hand, the dynamic coefficients, such as the rolling and yawing moment coefficients, are significantly affected by the rotational rate and the angle of attack. By defining the advance ratio ($\gamma_{advr}$) as in Equation (15), the dynamic coefficients of the roll and yaw moments can be easily formulated:

(15) $$\gamma_{advr}=\frac{d\cdot r}{2V_{T}}$$

where *r* denotes the rotation rate. First, consider *C_R_*, which is derived as:

(16) $$\begin{array}{ll} C_{R} & =C_{R\alpha }(\alpha )+\frac{d}{2V_{T}}pC_{Rp}(\alpha )+C_{Rr}(\alpha ,\gamma_{advr})\\ & ≃\frac{d}{2V_{T}}pC_{Rp}(\alpha )+C_{Rr}(\alpha ,\gamma_{advr}) \end{array}$$

In Equation (16), the static coefficient $C_{R\alpha }$(*α*) is approximated to zero because of the geometric symmetry of the disc. *C_Rp_* (*α*) is the roll damping derivative due to roll rate, which can be obtained as a constant. The roll moment coefficient due to the yaw rate, *C_Rr_*, which is a function of *α* and $\gamma_{advr}$, is mostly obtained through CFD analysis [2,3,4]. Next, consider the yawing moment coefficient *C_N_*, which is described as:

(17) $$\begin{array}{ll} C_{N} & =C_{N\alpha }(\alpha )+\gamma_{advr}\cdot C_{Nr}(r,V_{T})\\ & ≃\gamma_{advr}\cdot C_{Nr}(r,V_{T}) \end{array}$$

where $C_{N\alpha }\;$(*α*) can be also approximated to zero because of the rotating axis symmetry of the disc [21]. As a result, the yaw damping derivative *C_Nr_*, which is primarily caused by skin friction, becomes the most essential parameter for determining the yaw moment equation, subsequently influencing the whole flight characteristics of the disc.

Meanwhile, the force and moment equations are described in the *N*-frame and *B*-frame with the help of the Euler kinematics. First, the force equation is simply represented by Equation (18) in the *N*-frame with no control surfaces:

(18) $$\vec{F_{N}}=m_{T}(\vec{g_{N}}+\frac{d\vec{V_{N}}}{dt})$$

where ${\vec{V}}_{N}\left(V_{N},V_{E},V_{D}\right)$ is the velocity vector of the disc expressed in the *N*-frame, ${\vec{g}}_{N}\left(0,0,g\right)$ is the gravity vector, and *m_T_* is the total mass, which is the sum of the mass of the disc and the mass of the measurement module. Next, the moment is represented in the *B*-frame as shown below:

(19) $$\vec{M_{B}}=I\frac{d\vec{\omega }}{dt}+\vec{\omega }\times I\vec{\omega }$$

where $\vec{\omega }$ denotes the angular rate expressed in the *B*-frame, and *I* is the moment of the inertia matrix of the disc. Note that $\vec{\omega }={\left[p\;q\;r\right]}^{T}$. Now consider the aerodynamic force and moment represented in the *W*-frame, which are depicted as ${\vec{F}}_{W}^{A}$ and ${\vec{M}}_{W}^{A}$, respectively.

In Equation (20), note that the lateral force term and the vertical axis moment term are assumed to be zero, since the relative wind effect can be neglected in the sideslip force and yaw angular dynamics.

(20) $$\vec{F_{W}^{A}}=\left[\begin{array}{c} -D\\ 0\\ -L \end{array}\right],\;\vec{M_{W}^{A}}=\left[\begin{array}{c} R\\ M\\ 0 \end{array}\right]$$

**Figure A2:**
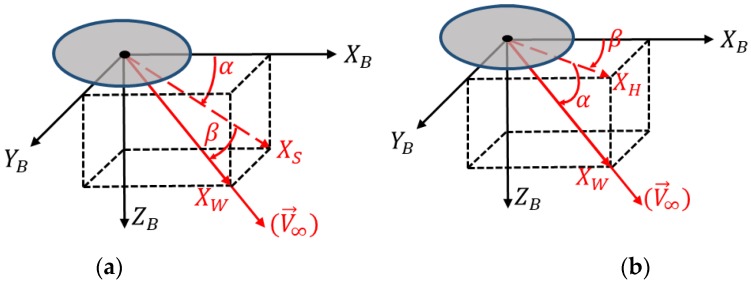
(**a**) Definition of the conventional aerodynamic angles, (**b**) Proposed definition of disc’s aerodynamic angles.

Finally, for solving the disc’s dynamic equations with the flight coefficients defined in the aerodynamic coordinate frame, coordinate transformations between frames are required [22]. In this study, the sequence of rotation in the transformation is newly defined for the ease of the dynamics analysis. Referring to Figure A2b, the force and moment in the *N*-frame and *B*-frame are described, respectively, as:

(21) $$\begin{array}{l} \vec{F_{N}^{A}}=C_{1}{}^{T}(\phi )C_{2}{}^{T}(\theta )C_{3}{}^{T}(\psi )C_{3}{}^{T}(\beta )C_{2}{}^{T}(-\alpha )\vec{F_{W}^{A}}\\ \vec{M_{B}^{A}}=\;C_{3}{}^{T}(\beta )C_{2}{}^{T}(-\alpha )\vec{M_{W}^{A}}+\left[\begin{array}{c} 0\\ 0\\ N \end{array}\right] \end{array}$$

where the Euler angles, angle of attack, and sideslip angle are employed. For an integrated analysis combining force and moment dynamics represented in different coordinate systems, the Euler angle’s propagation formula through the body rate is employed as shown below:

(22) $$\left[\begin{array}{c} p\\ q\\ r \end{array}\right]=\left[\begin{array}{c} 0\\ 0\\ \dot{\psi } \end{array}\right]+C_{3}\left(\left[\begin{array}{c} 0\\ \dot{\theta }\\ 0 \end{array}\right]+C_{2}\left[\begin{array}{c} \dot{\phi }\\ 0\\ 0 \end{array}\right]\right)$$

Finally, by combining ψ and β, which are both defined in the *B*-frame, Equation (21) can be further simplified into:

(23) $$\begin{array}{l} \vec{F_{N}^{A}}=C_{1}{}^{T}(\phi )C_{2}{}^{T}(\theta )C_{3}{}^{T}(\lambda )C_{2}(\alpha )\vec{F_{W}^{A}}\\ \vec{M_{B}^{A}}=\;C_{3}{}^{T}(\beta )C_{2}(\alpha )\vec{M_{W}^{A}}+\left[\begin{array}{c} 0\\ 0\\ N \end{array}\right] \end{array}$$

where λ≜β+ψ denotes the modified yaw angle between the *N*-frame and the newly defined *B*-frame, where the *x*-axis is aligned with the projected direction of the relative velocity vector.

In this study, the yawing moment coefficient was computed through the rotational rate estimated by the magnetic sensor mounted on the disc body experimentally. Thus, it could be readily defined in the *B*-frame. In contrast, other moment coefficients, such as pitching and rolling moment, were defined the wind coordinate system, since these coefficients can be efficiently calculated via CFD analysis in the *W*-frame [22].

### 4.3. Yaw Coefficient Computation and Application to Disc Dynamics Analysis

It is very important to estimate the yawing moment coefficient, since the rotational rate of the disc directly affects the overall flight trajectory and attitude stability. In this section, the yaw axis flight coefficient was derived using the proposed rate estimation method, then the resulting flight coefficient was applied to implement the flight simulator and obtain an accurate trajectory. Specifically, the rotational rate attenuation property based on the proposed estimation algorithm was investigated, then the derived the yaw damping derivative was applied to complete the simulator.

**Figure 10:**
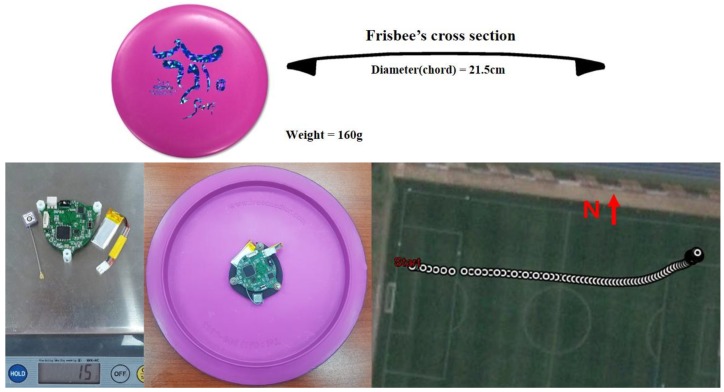
Disc and onboard sensor module for the field experiment.

Repeated throwing experiments were done at a soccer field using a driver disc attached to the miniaturized sensor module (shown in Figure 10). In the upper figure, the disc specifications are illustrated. In the lower left figure, the total weight of the embedded module alone and installed on the disc are shown. In each throwing, the whole flight trajectory was stored in the onboard memory and could be drawn on a digital map, as shown in Figure 10. The flying disc showed a trajectory that gradually curved to the left from the initial flight direction, and the flying distance was about 72 m. Repeated experimental data showing a similar flight trajectory pattern with a flight distance ranging between 70 m and 80 m were recorded. Simultaneously, for each experiment, the module stored the acceleration, angular velocity, and magnetic data from the IMU, and the velocity and position data from the GPS receiver.

Now consider again the yawing moment coefficient presented in Equation (17). By rearranging Equation (17), Equation (24) is obtained, which implies that the overall yawing moment coefficient is principally determined by the yaw damping derivative *C_Nr_*. The resulting aerodynamic yaw moment is equal to the yawing moment in the *z*-axis, as expressed in Equation (25). Thus, assuming an ideal disc rotation without perturbing factors such as precession, the damping derivative coefficient *C_Nr_* can be obtained by Equation (26), where it is expressed as a function of the relative velocity, yaw rate, and its time derivative:

(24) $$C_{N}=\frac{C_{Nr}(r,V_{T})\;rd}{2V_{T}}$$

(25) $$N=\frac{1}{16}\rho V_{T}C_{Nr}(r,V_{T})\;r\pi d^{4}$$

(26) $$C_{Nr}=\frac{16I_{zz}\dot{r}}{V_{T}r\rho \pi d^{4}}$$

**Figure 11:**
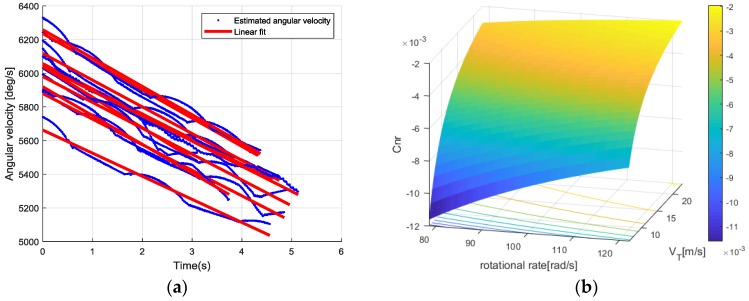
(**a**) Linearization of the flying disc’s angular velocity and (**b**) determination of two unknown values using 2D interporation and estimation of *C_Nr_*.

Finally, to calculate *C_Nr_*, $\dot{r}$ must be determined, according to Equation (26). Through repeated experiments, as shown in Figure 11, a number of rotational rate samples were accumulated. The blue dots in Figure 11a are the estimation results of the rotational rate over the flight period of each throw. In similar conditions of wind disturbance, all rate estimation results can be modeled with a linear attenuation slope. Therefore, the derivatives of the angular rate (i.e., angular acceleration) were approximated as constants by applying a linear fitting, which is shown as the red solid line in Figure 11a.

Meanwhile, as the angular acceleration is modeled as a constant, other variables, except for the angular rate and the disc’s relative velocity, were determined as constants in Equation (26). Thus, considering the disc’s flight ranges, the objective damping derivative could be summarized into a database form with respect to rate *r* and relative velocity *V_T_*. Through repeated experiments for generating the database, the bounds for angular rate and relative velocity ranged between 4500 and 7000 °/s and 6–23 m/s, respectively. With the defined ranges, the yaw damping derivative could be composed of a two-dimensional matrix as shown in Equation (27):

(27) $$C_{Nr}{\left(i,j\right)}_{k}=\frac{16I_{zz}\dot{r}(k)}{[(50(i-1)+4500)\frac{\pi }{180}](0.5(j-1)+6)\rho \pi d^{4}}$$

where *k* is the number of repeated experiments with integer *i* and *j*, (1 ≤ *i* ≤ 51, 1 ≤ *j*≤ 35). In Equation (27), note that the yaw rate has a separation node of 50 °/s, and the relative velocity has a separation node of 0.5 m/s. In addition, the yaw damping derivative matrix for each node could be averaged by the number of experiments, *N*, and be expressed as.

(28) $$C_{Nr}{\left(i,j\right)}_{avg}=(\sum_{k=1}^{N}C_{Nr}{\left(i,j\right)}_{k})/N$$

Figure 11b shows the 2-dimensional spline interpolation database of *C_Nr_* in terms of yaw rate and relative velocity. For the simulation, a piecewise continuous coefficient was needed, which was obtained from the pre-computed interpolation database between averaged *C_Nr_* matrix node values.

### 4.4. Flight Trajectory Analysis and Validation

**Figure 12:**
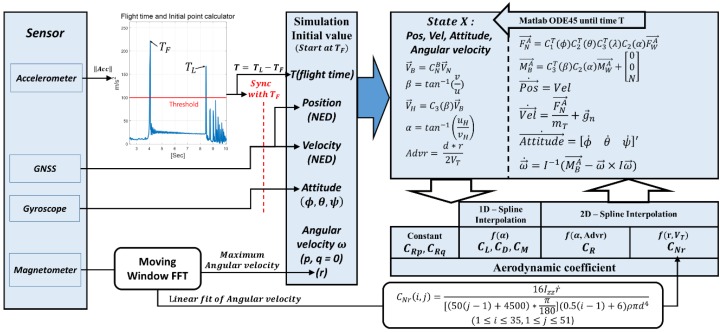
Structure of the disc flight simulator.

The aerodynamic coefficient was completed by reflecting the estimated yawing moment coefficients, and the flight dynamics model was constructed such that it could provide 12 state variables, including position, velocity, attitude, and angular rate. As noted previously, the yawing moment coefficient can be estimated using both methods based on an accelerometer and a magnetometer. In this paper, a simulator was designed to evaluate the performance for both cases. In both cases, the same initial values were provided, except for the estimated angular rate of the flying disc. Figure 12 shows the block diagram of the disc flight simulator with the parameters.

**Figure 13:**
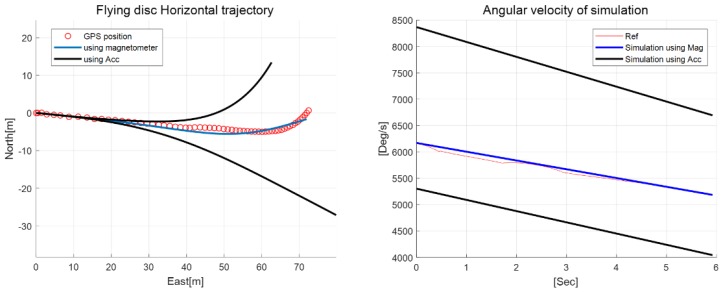
Simulation results of horizontal trajectory and angular rate (two estimation methods vs. analytic results of the simulator).

In the flight simulator, the initial position in the simulation is the virtual origin of the NED(North-East-Down) coordinate system. The initial velocity vector is expressed in the NED coordinate system, and the values are acquired from GPS velocity measurements and throwing angle. In the simulator, the attitude is defined as the Euler angle from the NED coordinate system order (i.e., roll–pitch–yaw). Thus, the initial attitude was set to −15° for roll, −17° for pitch, and 112° for yaw. The initial angular rates *p, q* were set to zero, and *r* was determined according to each estimated result of the accelerometer and magnetometer. Figure 13 shows the simulation results with the prescribed initial values. It can be seen that the blue solid line is closer than the black solid lines to the reference trajectory (red circle), which was given by the GPS measurements. Especially, the flying trajectory based on the accelerometer tended to diverge during the late periods of the flight through the accumulated yaw rate uncertainty. Consequently, magnetometer-based estimations provide a much improved prediction of the disc flight characteristics because of the precision and accuracy of the estimation of the yaw coefficient dynamics. A slight deviation from the reference trajectory resulted from the unmeasurable relative wind velocity and the higher-order aerodynamic coefficients.

## 5. Conclusions

This paper presents a new method for estimating the high rotation rate of a dynamic object by measuring the alternating geomagnetic field with an onboard low-cost magnetic sensor module. The proposed method takes advantage of the spectral analysis of the measured magnetic data in rotational motion with a moving-window approach. The new algorithm demonstrated superior estimation performance than the previous approach based on accelerometer measurements, since it is not directly affected by the misalignment errors occurring during sensor installation or other inertial perturbations. For a comparative study, an acceleration-based rate estimation method was revisited, which investigated a quantitative error boundary analysis. For performance verification of the algorithm, two embedded sensor modules were designed. A prototype module was manufactured for the comparison as a bulk-type module equipped with an ultrahigh 1-axis rate sensor, and a miniaturized module was produced for practical onboard testing. A reference rate table test demonstrated the accuracy of the proposed estimation method, despite computation burden and limited performance in the low rate region. To demonstrate a practical application, a flight test onboard the flying disc was performed. The estimated rate from the flight test was used to compute the flight coefficient governing the yaw moment dynamics. Consequently, the real disc flight test verified that the flight trajectory could be successfully predicted through the flight dynamics simulator, reflecting the estimated rate-related coefficient.

## Figures and Tables

**Figure A1:**
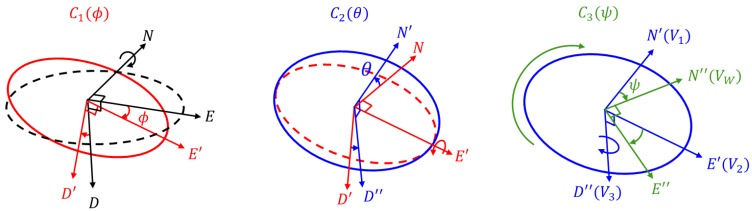
Flying disc’s Euler rotation order.

## Appendix A

Considering the inherent characteristics of the flying disc, a modified coordinate system is proposed for an insightful analysis of the disc flight dynamics. Four coordinate systems are introduced to properly represent the flying motion of the disc. First, the NED coordinate system is defined with the initial position of the disc as the origin, and the North, East, and Down as the corresponding axes. Unlike the previous disc studies, the NED coordinate system (i.e., *N*-frame) is employed as a base coordinate, which can facilitate the analysis using GPS data during the flight experiment. Second, the body-fixed coordinate system (i.e., *B*-frame) is defined with the flying disc’s center of mass as the origin. As known, *N*-frame and *B*-frame can be transformed via the Euler angle. Generally, the Euler angle rotation of an aircraft is done in the order of yaw–pitch–roll from the reference *N*-frame [23]. In contrast, the Euler rotation of a flying disc is better represented using the order of roll(*ϕ*)–pitch(*θ*)–yaw(*ψ*), since the disc shape has a perfect yaw symmetry during flight. Thus, the coordinate transformation matrix with the new rotation order is given by $C_{3}(\psi )C_{2}(\theta )C_{1}(\phi )$ where:

(A1) $$C_{1}\left(•\right)=\left[\begin{array}{ccc} 1 & 0 & 0\\ 0 & \cos\left(•\right) & \sin\left(•\right)\\ 0 & -\sin\left(•\right) & \cos\left(•\right) \end{array}\right],\;C_{2}\left(•\right)=\left[\begin{array}{ccc} \cos\left(•\right) & 0 & -\sin\left(•\right)\\ 0 & 1 & 0\\ \sin\left(•\right) & 0 & \cos\left(•\right) \end{array}\right],\;C_{3}\left(•\right)=\left[\begin{array}{ccc} \cos\left(•\right) & \sin\left(•\right) & 0\\ -\sin\left(•\right) & \cos\left(•\right) & 0\\ 0 & 0 & 1 \end{array}\right]$$

Figure A1 shows the rotation sequence of the flying disc [24]. While the disc rotates about the *D″*-axis after the roll–pitch rotation, the shape viewed from the *z*-axis does not change because of its rotational symmetry. This implies that the flying disc’s shape observed from an arbitrary viewpoint is determined simply by performing the roll and pitch rotation from the *N*-frame.

For developing flight dynamics considering the aerodynamic wind effect, the wind-axis coordinate system (*W*-frame) is generally employed. As shown in Figure A2a, the W-frame can be derived from the *B*-frame using the angle of attack (α) and the sideslip angle (β). Typically, the stability-axis coordinate system (*S*-frame) is employed as an intermediate coordinate system between the *B*-frame and the W-frame in most flight dynamics analysis [21]. Note that the *x* axis of the *S*-frame is defined using the sideslip angle from the *x*-axis of the *W*-frame, which corresponds to the relative wind vector direction. However, in the case of disc flight, the sideslip angle changes periodically from 0 to 360° as a result of rotation. In particular, when the relative wind exists in the *Y–Z* plane of the flying disc (i.e., $\beta =90^{{}^{\circ}},\;270^{{}^{\circ}}$), the angle of attack equals 90° by definition. Thus, employing the S-frame may cause a singularity problem [21,24]. To resolve this, this paper introduces an alternative interpretation of the aerodynamic angle, as shown in Figure A2b. First, the heading-axes coordinate system (*H*-frame) is newly defined by rotating the B-frame using the sideslip angle, where its *x*-axis is denoted by *X_H_*. Then, the wind-axis coordinate system can be easily represented through the angle of attack(α), which coincides with the rotation angle between the *x*-axis of the *H*-frame and the relative wind-axis. Employing the *H*-frame as a new intermediate coordinate system is always possible because of the shape of the flying disc, since the chord length in any angle of sideslip by rotation does not change.

With the coordinate system definition of the *H*-frame, the aerodynamic angles of the disc can be derived as shown in the following equation:

(A2) $$\begin{array}{l} \beta =\tan^{-1}(\frac{v}{u})\\ \vec{V_{H}}=C_{3}(\beta )\vec{V_{B}}\\ \alpha =\tan^{-1}(\frac{w_{H}}{u_{H}}) \end{array}$$

where ${\vec{V}}_{B}={\left[u\;v\;w\right]}^{T}$ represents the velocity vector of the origin in the *B*-frame, ${\vec{V}}_{H}={\left[u_{H}\;v_{H}\;w_{H}\right]}^{T}$ represents the coordinated transformed velocity vector in the *H*-frame. With the defined *H*-frame, the angle of attack can be obtained via Equation (A2) without a singularity problem.
